# Physician Preferences for Universal Routine Depression Screening for Adolescents in Primary Care

**DOI:** 10.1001/jamanetworkopen.2025.45361

**Published:** 2025-11-25

**Authors:** Tran T. Doan, Davene R. Wright, Melissa DeJonckheere, David W. Hutton, Kristin N. Ray, Lisa A. Prosser

**Affiliations:** 1 Department of Health Systems, Management and Policy, Colorado School of Public Health, University of Colorado Anschutz Medical Campus, Aurora; 2 Department of Population Medicine, Harvard Pilgrim Health Care Institute and Harvard Medical School, Boston, Massachusetts; 3 Department of Family Medicine, University of Michigan Medical School, Ann Arbor; 4 Department of Health Management and Policy, University of Michigan School of Public Health, Ann Arbor; 5 Department of Pediatrics, University of Pittsburgh School of Medicine, Pittsburgh, Pennsylvania; 6 Susan B. Meister Child Health Evaluation and Research Center, University of Michigan Medical School, Ann Arbor

## Abstract

**Importance:**

Universal adolescent depression screening is recommended as routine primary care, but it is unclear how best to implement it. Systematic evaluation of physician preferences can support optimal screening implementation.

**Objective:**

To assess primary care physicians’ preferences for different attributes of a universal adolescent depression screening strategy.

**Design, Setting, and Participants:**

In this survey study, a discrete choice experiment was administered to a US physician panel maintained by Qualtrics. Five attributes were identified from prior qualitative work: screening modality, screening location, screening completion time, missed depression cases, and clinical examination time, each with 2 to 3 levels. The survey presented 13 discrete choice questions and physician characteristics’ questions. The survey was pretested through cognitive debriefings, piloted to an independent sample, and fielded to the final sample from April to June 2024.

**Main Outcomes and Measures:**

Physicians’ preference and importance coefficients for different screening strategies using time to assess willingness to make trade-offs for changes in attribute levels were estimated. Data were analyzed using conditional logit and latent class models.

**Results:**

Among the 181 physician respondents (96 males [53.0%]), 90 (49.7%) were in urban settings, 112 (61.9%) were in a pediatrics primary care specialty, and 68 (37.6%) were in private practice. Conditional logit analyses showed that respondents preferred the least missed depression cases (59.6% importance), shortest clinical examination time (21.0% importance), 3-minute screening time (12.9% importance), private area provision (3.8% importance), and electronic modality (2.7% importance). Physicians reported their willingness to spend 37.3 minutes (95% CI, 32.1-42.5 minutes) during examinations per patient to reduce missed diagnoses from 10% to 5%. In the latent class analysis, 3 subgroups were identified. The diagnostic accuracy-sensitive group (n = 66) prioritized the top important attribute, missed depression cases (75.1% importance), more than other groups. The clinic time-sensitive group (n = 33) prioritized shortening examination time (35.2% importance) more than other groups. The screener type-specific group (n = 82) prioritized 3-minute screening (25.8% importance) and electronic modality (10.2% importance) more than other groups.

**Conclusion and Relevance:**

In this survey study, primary care physicians preferred identifying adolescent depression accurately and shortening well-child examination time. Health systems and payers should consider these preferences for accuracy and efficiency by physicians, who are strongly positioned to identify adolescent depression early and to implement screenings. Further research is needed to better understand family and administrative staff preferences regarding pediatric integrated behavioral health care.

## Key Points

**Question:**

What are the quantitative preferences of primary care physicians for different attributes of a routine universal adolescent depression screening strategy?

**Findings:**

In this survey study containing a discrete choice experiment among 181 physician respondents, top preferences were (1) accurately identifying depression cases and (2) shortening clinical appointment times. Physicians also preferred brief electronic administration and provision of a private screening area, and they reported willingness to spend 37 minutes per patient to reduce missed diagnoses (false negatives) from 10% to 5%.

**Meaning:**

These findings suggest that health systems and payers should consider physicians’ preferences for accuracy and efficiency when determining strategies to implement national guidelines on universal depression screening during annual well-child care visits.

## Teaser

This survey study assesses primary care physicians’ preferences for attributes of a universal routine depression screening strategy for adolescents.

## Introduction

Approximately one-fifth of youths aged 12 to 17 years in the US had a major depressive episode in 2022.^1^ Only half of adolescents with major depression (hereinafter depression) are diagnosed before reaching adulthood.^2^ Less than half of adolescent patients who are diagnosed with depression receive the necessary treatment^1^ and follow-up care.^3^ Depression, especially untreated depression, is a predictor of suicide, the second-foremost cause of adolescent deaths.^4^ About three-fourths of adult mental health disorders had onset during childhood, with increasing difficulty in treating them the longer the treatment lapse.^5^

Pediatricians and other primary care physicians are strongly positioned to identify and initiate treatment for adolescent depression.^2,6^ Since 2018, the American Academy of Pediatrics recommends that pediatricians perform annual universal depression screenings in youths aged 12 years and older.^2^ Rates of universal adolescent depression screening remain variable (ranging from 0% to 82%) across health systems and are inconsistent over time.^7,8,9,10,11,12^ Physicians face substantial implementation barriers (cost constraints, confidentiality concerns, and time barriers)^13,14,15,16^ and must weigh all national directives against practical constraints of what can be realistically accomplished during well-child examinations.^17^

Universal adolescent depression screening is recommended as part of routine primary care, but it is unclear how best to implement it with multiple potential features at odds with each other (comprehensiveness vs efficiency). Understanding physician preferences can help health systems and payers design optimal features of a universal screening program to improve clinician acceptability^18^ and effectively reach adolescent patients in need.

A conjoint analysis developed for physician respondents may help elucidate systematic preferences to guide successful screening strategies. Conjoint analysis is an analytic tool used to elicit quantitative preferences of decision-makers for different program features.^19^ Examples of conjoint-based health applications include deciding adult depression treatments,^20,21^ childhood combination vaccines,^22^ pediatric medical homes,^23^ and childhood obesity treatments.^24^ Eliciting physicians’ preferences may help understand their decision-making when weighing competing priorities^17^ and universal screening options.^13,25,26^

This study aimed to elicit physician preferences and time trade-offs for different attributes of annual universal adolescent depression screening in pediatric primary care. We sought to assess which key attributes of a universal screening program were associated with an increased likelihood of screening among physicians practicing in the US. We hypothesized that preferences would differ by key features of universal screening strategies and distinct physician characteristics. Findings can be used by the medical community, health systems, insurance payers, and national bodies to consider which aspects of universal adolescent depression screening programs should be prioritized and resourced.

## Methods

### Overview

In this survey study, we conducted a discrete choice experiment (DCE), a type of conjoint analysis,^19^ to quantify the strength of preferences across a national physician sample for different screening attributes. In a DCE, respondents are shown a series of hypothetical options and asked to choose which option would increase the probability of engaging in screening (eTable 1 in Supplement 1). By analyzing respondents’ choices using statistical models, preference coefficients can be estimated to indicate the extent to which each attribute was associated with the screening decision.^27,28,29^ Preference findings could be leveraged to develop an optimal screening program incorporating physician preferences. The University of Michigan institutional review board and the University of Pittsburgh institutional review board determined that this study was exempt from review because it did not constitute human participant research. Informed consent was obtained. This study followed guidelines from the International Society for Pharmacoeconomics and Outcomes Research Good Research Practices for Conjoint Analysis Task Force^19,30^ and the American Association for Public Opinion Research (AAPOR) reporting guideline (eTable 2 in Supplement 1).

### Sampling and Data Collection

Respondents were sampled from April to June 2024 through a national physician panel maintained by Qualtrics, a survey research firm. Using Qualtrics, survey data were collected efficiently and shared without identifiable information. Respondents were invited by email and underwent a validation process to verify themselves as medical professionals. Respondents were eligible if they practiced in pediatrics, family medicine, or internal medicine and regularly saw patients aged 12 to 21 years as part of clinical practice.

Qualtrics was paid $58 for each high-quality, completed physician survey response. Qualtrics excluded inadequate quality responses and replaced these respondents by inviting a new participant, and therefore, there were no missing responses. Responses were determined to be of inadequate quality if completed in less than 2 minutes, a pattern was detected across questions, percentages of attribute importance were illogical, or the direction of attribute preferences was illogical. Consistent with the AAPOR reporting guideline, because the sample was recruited from an opt-in, nonprobability panel, the participation rate cannot be reported because the sampling frame was unknown.^31^

### Attributes and Levels

Attributes described the characteristics of depression screening. Each attribute was characterized by 2 to 3 attribute levels. Attributes and levels were derived using a sequential mixed methods approach^32,33^ and informed by our prior qualitative findings.^25^ Final attributes included screening modality, screening location, screening completion time, missed depression cases, and clinical examination time (Table 1).

**Table 1.** Attributes and Levels for Universal Annual Screening of Adolescent Depression in Primary Care

Attribute	Level	Description
Screening modality	Paper; electronic	Depression screener version can be administered using paper or electronic forms.
Screening location	No private area; provision of a private area	Adolescent patients can be provided with a private area to complete the screener alone.
Screening completion time	10 min; 3 min; 30 s	Depression screening tools vary in terms of how long they take to fill out.
Missed depression cases	25%; 10%; 5%	Screening tools vary in terms of how good they are at identifying accurate depression cases.
Clinician examination time	60 min; 45 min; 30 min	This is the average length of time that a primary care clinician is allotted to spend with adolescent patients, including the scheduled appointment time and allotted documentation or administrative time.

### Survey Instrument

The survey instrument was programmed in Sawtooth version 9.15.4 (Sawtooth Software Inc, Provo, Utah) (eAppendix in Supplement 1). The survey included a brief introduction to the US context of universal adolescent depression screening recommendations and descriptions of each attribute and its levels.

Respondents were shown 13 discrete choice questions, each presenting a panel of 2 hypothetical choice profiles and an opt-out third option. While completing the DCE, respondents were instructed to imagine which option would best increase the adolescent depression screening rate within their primary care practice and to assume their practice had adequate resources for providing referral options and other mental specialty care, as this study evaluated depression identification and diagnosis, rather than treatment or management.

Discrete choice profiles included efficiently designed combinations of attribute levels. These profiles were generated using a fractional factorial design with a balanced overlap of 2353 possible questions to optimize statistical power while reducing participant burden.^19,30,34^ Implausible combinations of choice profiles were excluded (10-minute screening time was prohibited from appearing with 25% missed depression, and 30-second screening time was prohibited from appearing with 5% missed depression).

Cognitive debriefings were conducted among pediatricians (n = 5) to confirm attributes and to pretest the survey framing and questions. Of these participants, 4 self-identified as female, 4 as White, and 1 as Asian. The survey was then pilot tested with an independent physician sample (n = 50) from October to November 2021 to estimate response times and logical preferences. Based on both cognitive debriefings and pilot testing, survey introduction language was revised to be relevant to primary clinical practice, and a choice practice question and 2 demographic questions (region, specialty) were added. The survey concluded with sociodemographic questions about gender, race and ethnicity, geographic setting and region, primary care specialty, and practice setting. The survey’s race and ethnicity categories were self-reported and included American Indian or Alaska Native; Asian or Asian American; Black or African American; Hispanic, Latino, or of Spanish origin; Middle Eastern; Native Hawaiian or Other Pacific Islander; White; or mixed race or ethnicity. Race and ethnicity were included in the survey to understand how they may shape decision-making preferences around implementing adolescent depression care in primary care across contexts.

### Implementation Framework

The updated Consolidated Framework for Implementation Research (CFIR)^35^ organizes determinants of universal adolescent depression screening to help improve implementation efforts of proven interventions.^36^ Incorporating physician preferences could improve screening implementation across the 5 domains of the CFIR: (1) outer setting (policy, incentives), (2) inner setting (workflow, priorities), (3) innovation (design, cost), (4) individuals (preferences, acceptability), and (5) process (planning, training). Implementation was operationalized as the extent to which screening activities were routinely delivered in adolescent primary care. Attributes were selected to reflect different screening options that could be implemented or traded off against each other.

### Outcome Measures

We reported a part-worth preference coefficient for each attribute level over the sample. Coefficients represented the extent to which physicians preferred certain attribute levels relative to others. The higher the coefficient, the more the level was preferred, and the smaller the coefficient, the less the level was preferred. Coefficients were estimated using conditional logit and latent class statistical analyses.^37^

We reported a relative importance score for each attribute. The importance score can be interpreted as the degree to which an attribute changed decisions relative to others and was calculated as follows: for each attribute, the difference between coefficients of attribute levels was divided by the sum of differences between coefficients for all attributes and multiplied by 100.

We reported a willingness to trade time for each attribute level. The value attribute was minutes spent by physicians per patient during clinical examinations and was chosen because it was considered meaningful to physicians in cognitive debriefings. The willingness to trade time for implementing a different attribute level was calculated by dividing the preference coefficient of that specific attribute level by the linear preference coefficient of the mean clinical examination time (equation in next section).^37,38^

### Statistical Analysis

Conditional logit analysis was used to relate the probability of choosing a screening program (*Pr*[choice]) as a function of specific attribute levels (Attribute_x_) for that program. Choice likelihood was represented as *Y* = 1 if a particular profile was chosen and *Y* = 0 if not chosen. The linear probability function was estimated as: *Pr*(choice) = *Pr*(*Y* = 1) = β_0_ + β_1_ × Attribute_1_ + β_2_ × Attribute_2_ + β_3_ × Attribute_3_.

β*_i_* Coefficients were interpreted as part-worth preference probabilities. Conditional logit modeling was used to calculate mean coefficients over an entire sample, but it cannot account for preference heterogeneity (each respondent may have different preferences).^37^

To examine preference heterogeneity, latent class analysis was used to distinguish physician groups with similar preferences.^37^ This model assumed that attributes and levels can have heterogeneous effects on choices across derived groups.^37^ A latent class model can estimate group membership and part-worth probabilities for each derived group. Within each group, preference coefficients were calculated using conditional logit analysis. Statistical analyses were performed using Sawtooth, version 9.15.4, and 2-sided *P* < .05 was the threshold for statistical significance.

## Results

### Study Respondents

The final sample included 181 physician respondents (83 females [45.9%] and 96 males [53.0%]) who completed high-quality survey responses (Table 2). An additional 117 responses were completed but determined to be of inadequate quality based on a priori rules and were therefore not included in the data analysis. There were 90 participants (49.7%) in urban settings, 91 (50.3%) in the South, 112 (61.9%) in a pediatrics specialty, and 68 (37.6%) in private practice. In terms of race and ethnicity, 5 (2.8%) were American Indian or Alaska Native; 14 (7.7%) were Asian or Asian American; 11 (6.1%) were Black or African American; 8 (4.4%) were Hispanic, Latino, or of Spanish origin; 5 (2.8%) were Middle Eastern; 1 (0.6%) was Native Hawaiian or Other Pacific Islander; 134 (74.0%) were White; and 3 (1.7%) were of mixed race or ethnicity.

**Table 2.** Primary Care Physician Respondent Characteristics for the Total Sample and Latent Groups

Characteristic	Group, No. (%)				*P* value^a^
	Full sample	Diagnostic accuracy-sensitive	Clinic time-sensitive	Screener type-specific	
Sample size	181 (100.0)	66 (36.5)	33 (18.2)	82 (45.3)	NA
Gender					
Female	83 (45.9)	33 (50.0)	19 (57.6)	31 (37.8)	.39
Gender variant or gender nonconforming	1 (0.6)	0	0	1 (1.2)	
Male	96 (53.0)	33 (50.0)	14 (42.4)	49 (59.8)	
Prefer not to disclose	1 (0.6)	0	0	1 (1.2)	
Race and ethnicity					
Hispanic, Latino, or of Spanish origin	8 (4.4)	2 (3.0)	1 (3.0)	5 (6.1)	.34
Non-Hispanic					
American Indian or Alaska Native	5 (2.8)	0	1 (3.0)	4 (4.9)	
Asian or Asian American	14 (7.7)	3 (4.5)	2 (6.1)	9 (11.0)	
Black or African American	11 (6.1)	3 (4.5)	2 (6.1)	6 (7.3)	
Middle Eastern	5 (2.8)	2 (3.0)	2 (6.1)	1 (1.2)	
Native Hawaiian or Other Pacific Islander	1 (0.6)	0	0	1 (1.2)	
White	134 (74.0)	56 (84.8)	25 (75.8)	53 (64.6)	
Mixed race or ethnicity	3 (1.7)	0	0	3 (3.7)	
Geographic setting					
Urban	90 (49.7)	29 (43.9)	12 (36.4)	49 (59.8)	.13
Suburban	77 (42.5)	30 (45.5)	19 (57.6)	28 (34.1)	
Rural	11 (6.1)	6 (9.1)	2 (6.1)	3 (3.7)	
Urban and suburban	2 (1.1)	0	0	2 (2.4)	
None of these settings or did not select setting	1 (0.6)	1 (1.5)	0	0	
Geographic region					
Northwest	35 (19.3)	13 (19.7)	8 (24.2)	14 (17.1)	.02
Midwest	34 (18.8)	6 (9.1)	11 (33.3)	17 (20.7)	
South	91 (50.3)	42 (63.6)	9 (27.3)	40 (48.8)	
West	21 (11.6)	5 (7.6)	5 (15.2)	11 (13.4)	
Primary care specialty					
Pediatrics	112 (61.9)	51 (77.3)	29 (87.9)	32 (39.0)	<.001
Family medicine	57 (31.5)	13 (19.7)	4 (12.1)	40 (48.8)	
Internal medicine	12 (6.6)	2 (3.0)	0	10 (12.2)	
Practice setting					
Academic hospital only	58 (32.0)	16 (24.2)	6 (18.2)	36 (43.9)	.001
Community health center only	35 (19.3)	9 (13.6)	6 (18.2)	20 (24.4)	
Private practice only	68 (37.6)	31 (47.0)	21 (63.6)	16 (19.5)	
Academic hospital and community health center	10 (5.5)	5 (7.6)	0	5 (6.1)	
Community health center and private practice	2 (1.1)	2 (3.0)	0	0	
All 3 practice settings	7 (3.9)	3 (4.5)	0	4 (4.9)	
None of these settings or did not select setting	1 (0.6)	0	0	1 (1.2)	

Abbreviation: NA, not applicable.

^a^ Calculated using the Pearson χ^2^ test.

### Physician Preferences for Universal Adolescent Depression Screening Strategies

Per the coefficient (SE) estimates, physician respondents preferred achieving the least missed depression cases (0.78 [0.06], *P* < .001; 59.6% importance), offering a 30-minute appointment time (0.31 [0.04], *P* < .001; 21.0% importance), completing a 3-minute screener (0.19 [0.04], *P* < .001; 12.9% importance), providing a private area for adolescent patients to screen alone (0.06 [0.03], *P* = .05; 3.8% importance), and administering an electronic modality (0.04 [0.03], *P* = .15; 2.7% importance) (Table 3). Respondents opted out of 143 of 2353 (6.1%) choice questions.

**Table 3.** Primary Care Physicians’ Part-Worth Preference Coefficients, Importance Scores, and WTT in Minutes for Attributes and Levels of Universal Adolescent Depression Screening (N = 181)

Attribute	Coefficient (SE)^a^	*P* value^b^	Importance, %^c^	WTT (95% CI), min^d^
Screening modality				
Paper	−0.04 (0.03)	.15	2.7	−1.9 (−4.6 to 0.7)
Electronic	0.04 (0.03)	.15		1.9 (−0.7 to 4.6)
Screening location				
No private area	−0.06 (0.03)	.05	3.8	−2.7 (−5.4 to 0)
Provision of a private area	0.06 (0.03)	.05		2.7 (0 to 5.4)
Screening completion time				
10 min	−0.20 (0.05)	<.001	12.9	−9.3 (−14.1 to 4.5)
3 min	0.19 (0.04)	<.001		9.2 (5.0 to 13.4)
30 s	0 (0.05)	.98		0.1 (−4.9 to 5.1)
Missed depression cases, %				
25	−1.01 (0.06)	<.001	59.6	−48.0 (−53.5 to 42.6)
10	0.22 (0.04)	<.001		10.7 (6.7 to 14.7)
5	0.78 (0.06)	<.001		37.3 (32.1 to 42.5)
Clinical examination time, min				
60	−0.31 (0.04)	<.001	21.0	NA
45	0 (0.04)	.99		NA
30	0.31 (0.04)	<.001		NA
Opt-out	−1.97 (0.09)	<.001	NA	NA

Abbreviations: NA, not applicable; WTT, willingness to trade time.

^a^ Results were estimated using effects coding in conditional logit statistical analysis. Preference coefficients represented the extent to which physicians preferred certain attribute levels relative to others. A positive coefficient indicated a positive preference, and a negative coefficient indicated a negative preference. The higher the coefficient, the more the level was preferred, and the smaller the coefficient, the less the level was preferred.

^b^ Calculated using the 95% significance test.

^c^ Calculated as follows: for each attribute, the difference between the utilities of its levels was divided by the sum of the differences between the utilities for all of the attributes and multiplied by 100.

^d^ Calculated by dividing the preference coefficient for a specific attribute level by the preference coefficient for the clinical examination time, assuming linearity. The linear coefficient for mean clinical examination minutes was 0.02.

A visual depiction of coefficients may be useful to show the relative importance of each attribute level compared with one another (Figure). Of all levels, the top preferred level was reducing the number of missed depression cases, and the second preferred level was the shortest clinical examination time of 30 minutes.

**Figure.**
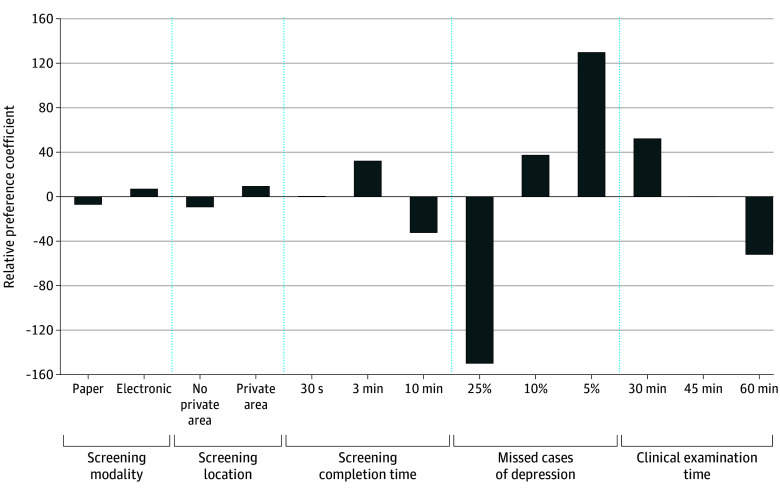
Relative Preferences for Attribute Levels of Universal Adolescent Depression Screening by Primary Care Physicians The y-axis represents the relative preference coefficients of each attribute level compared with all other attribute levels, as determined by the conditional logit analysis. Coefficients above the 0 reference line indicate a positive preference for that level. Coefficients below the 0 reference line indicate that the level was not preferred. The higher the coefficient, the higher the extent to which the level was preferred and vice versa. Coefficients were rescaled using zero-centered differences, which transformed the raw utilities for each attribute such that their sum equals 0. This approach centered the utility values around 0, making it easier to interpret the relative desirability or aversion associated with different attribute levels.

We performed an additional hierarchical bayesian analysis, accounting for within-respondent clustering, which yielded results broadly consistent with conditional logit estimates with minor differences for missed depression cases, indicating greater sensitivity for this attribute across respondents (results not shown). Physicians would be willing to spend 37.3 minutes (95% CI, 32.1-42.5 minutes) during clinical examination time per screened patient to reduce missed depression cases from 10% to 5% (Table 3). Physicians would spend 1.9 minutes (95% CI, −0.7 to 4.6 minutes) implementing an electronic screener, 2.7 minutes (95% CI, 0 to 5.4 minutes) providing a private screening area, and approximately no time (0.1 minute [95% CI, −4.9 to 5.1 minutes]) implementing a 30-second screener (Patient Health Questionnaire-2 [PHQ-2]).^39^

### Variations in Preferences

The latent class analysis yielded 3 distinct groups, selected for a better model fit based on Akaike Information Criterion or Bayes Information Criterion estimates comparing 2 to 5 possible groups. All 3 groups weighed missed depression cases as the top important attribute (Table 4).

**Table 4.** Primary Care Physicians’ Relative Preference Coefficients, Importance Scores, and Willingness to Trade Time in Minutes for Attributes and Levels of Universal Adolescent Depression Screening

Attribute	Full sample (N = 181)		Group					
			Diagnostic accuracy-sensitive (n = 66)		Clinic time-sensitive (n = 33)		Screener type-specific (n = 82)	
	Coefficient^a^	*P* value^b^	Coefficient^a^	*P* value^b^	Coefficient^a^	*P* value^b^	Coefficient^a^	*P* value^b^
Screening modality								
Paper	−6.8	.15	−2.3	.61	−3.3	.60	−25.4	.04
Electronic	6.8	.15	2.3	.61	3.3	.60	25.4	.04
Screening location								
No private area	−9.4	.05	−4.8	.31	−27.0	<.001	2.5	.84
Provision of a private area	9.4	.05	4.8	.31	27.0	<.001	−2.5	.84
Screening completion time								
10 min	−32.5	<.001	−13.4	.10	−29.5	.005	−68.8	.003
3 min	32.2	<.001	20.1	.004	28.2	.004	60.1	.002
30 s	0.3	.98	−6.7	.41	1.3	.91	8.7	.71
Missed depression cases, %								
25	−167.7	<.001	−206.4	<.001	−120.7	<.001	−145.2	<.001
10	37.4	<.001	37.1	<.001	35.5	<.001	64.9	.001
5	130.3	<.001	169.2	<.001	85.2	<.001	80.3	.001
Clinical examination time, min								
60	−52.5	<.001	−35.2	<.001	−95.2	<.001	−46.0	.01
45	0.1	.98	−6.2	.39	14.3	.12	2.2	.91
30	52.3	<.001	41.4	<.001	80.9	<.001	43.8	.02
Opt-out	−329.3	<.001	−352.7	<.001	16.6	.08	−1440.6	<.001
Importance, %^c^								
Screening modality	2.7	NA	0.9	NA	1.3	NA	10.2	NA
Screening location	3.8	NA	1.9	NA	10.8	NA	1.0	NA
Screening completion time	12.9	NA	6.7	NA	11.5	NA	25.8	NA
Missed depression cases	59.6	NA	75.1	NA	41.2	NA	45.1	NA
Clinical examination time	21.0	NA	15.3	NA	35.2	NA	17.9	NA

Abbreviation: NA, not applicable.

^a^ Results were estimated using effects coding in conditional latent class analysis. Preference coefficients represented the extent to which physicians preferred certain attribute levels relative to others. A positive coefficient indicated a positive preference, and a negative coefficient indicated a negative preference. The higher the coefficient, the more the level was preferred, and the smaller the coefficient, the less the level was preferred. Coefficients were rescaled using zero-centered differences, transforming raw utilities to a scale in which the mean difference between the smallest and largest levels across all attributes equaled 100. SEs corresponding with rescaled coefficients using zero-centered differences were not reported due to the absence of covariance estimates required to correctly transform SEs after rescaling.

^b^ Calculated using the 95% significance test.

^c^ The relative importance score was calculated as follows: for each attribute, the difference between the utilities of its levels was divided by the sum of the differences between the utilities for all of the attributes and multiplied by 100.

The diagnostic accuracy-sensitive group (66 [36.5%]) prioritized reducing missed diagnoses (75.1% importance) even more than other groups. For this group, respondents were more likely to practice in the South, in pediatrics or in family medicine, or in academia or in private practice (Table 2).

The clinic time-sensitive group (33 [18.2%]) prioritized shortening examination time (35.2% importance) more than others. This group had its highest proportion of respondents practicing in pediatrics and private practice (Table 2).

The screener type-specific group (82 [45.3%]) prioritized a 3-minute completion time (25.8% importance), approximating the widely adopted screener, PHQ-9,^40^ and an electronic modality (10.2% importance) more than other groups. For this group, respondents were more likely to be diverse regarding primary care specialties and practice in academic or community settings (Table 2).

## Discussion

In this survey study, we administered a DCE to a national physician sample to evaluate different implementation features of universal routine depression screening in adolescent primary care associated with increased screening likelihood. The findings suggest that top important attributes were reducing missed depression cases (false negatives) and avoiding excess well-child appointment time. Physicians also preferred a 3-minute screening time, a private screening area, and an electronic administration.

The mental health crisis among children has escalated concerns from primary care practitioners. The American Academy of Pediatrics, the American Academy of Child and Adolescent Psychiatry, and the Children’s Hospital Association declared a national state of emergency in children’s mental health in 2021.^41^ Many practitioners urge the integration of primary care behavioral health services,^42,43^ including implementing routine universal depression screenings for youths aged 12 years and older.^2,44^

According to physician preferences, our findings suggest that national bodies, insurance payers, pediatric health systems, and individual primary care practices should prioritize strategies that enhance diagnostic accuracy and address time constraints, which could improve the national screening rate. In concert with physician preferences, if feasible, practices should provide adolescent patients with an in-office private area to complete screenings independently of caregivers. Depending on a practice’s operational capacity, electronic screening may be completed in-office using a tablet or by patients at home alone before visits.^25^ However, our study did not distinguish between these 2 modalities to reduce participant burden.

Physicians may value flexibility in scheduling to ensure that adequate attention is paid to patients’ mental health concerns.^25^ Although physicians in our study preferred shorter visit times, qualitative interviews suggested that they desire longer visits but were constrained by inadequate financial incentives and demands for high patient volumes.^25,45^ Aligning reimbursement with value-based payment or integrated behavioral health care coverage may help reconcile time constraints. While physicians reported that they were willing to spend a mean 37 minutes to improve diagnostic accuracy, some of this additional time might be used to assess comorbidities (particularly with anxiety or eating disorders) or suicidality (requiring involvement from caregivers).^25^ Physicians preferred the PHQ-9 as the primary screening tool if selecting only one. However, inconclusive screening results may warrant follow-up with a more comprehensive instrument, such as the Children’s Depression Rating Scale, in which administration can take up to 45 minutes when interviewing both the child and caregiver.^46^

Primary care physicians are often on the front lines of identifying, diagnosing, and caring for adolescent patients with depression. Physician perspectives should be considered to promote the uptake of universal routine adolescent depression screenings.^13,25,26^ Most pediatricians think that they should identify young patients with mental health issues.^13,14^ Qualitative research found that pediatricians perceived that addressing time constraints, mental health workforce shortages, and training for accurate diagnosis and treatment could be helpful to improve pediatric depression screening rates.^14,25,26^ Past research reported the effectiveness of implementing universal depression screening in adolescent primary care,^7,42,43,47,48,49^ but quantitative preferences had been previously unknown. Quantitative preferences around depression treatment and knowledge attainment^50^ have been clarified for adults,^20,21,51,52,53,54,55^ adolescents and young adults,^56,57,58,59^ parents,^60,61^ and mental health specialists^62,63,64^ but not for physicians or around depression screening.

Regarding adult-based screenings, a pilot study of participatory approaches to implementing universal depression screening found that primary care clinical and administrator participants considered tablet-based administration to be feasible and acceptable, although integrating it into clinical workflows remained a challenge.^65^ A quality improvement initiative found that enhancing the electronic health record workflow, providing clinician and staff training, and standardizing data monitoring processes could improve the implementation of depression screening for adults.^66^ To effectively promote depression screening among multicultural patients, it is crucial to strengthen efforts in clinician team engagement and care coordination.^67^

This discrete choice study provides the first, to our knowledge, quantitative estimates of physician preferences to inform decision-making regarding universal depression screening and adolescent mental health care. Primary care offices and health systems should adopt distinct, relevant practices tailored to their individual clinical contexts to implement routine screenings effectively.^7,42,43^ Different physician groups might prefer different screening programs, whose medical decision-making may be associated with their demographic or practice characteristics^64^ or current abilities, perceptions, or biases.^68,69^ Our study found that physicians placed varying importance on diagnostic accuracy, clinical examination time, and screener type. For example, the clinic time-sensitive group prioritized shorter well-examination times and was more likely to practice in private settings, potentially to support higher patient volumes. Compared with this group, the diagnostic accuracy-sensitive and the screener type-specific groups had higher proportions of respondents practicing in academic settings, in which financial structures and resources differ, and more comprehensive evaluations may be emphasized given the teaching environment. These variations in priorities should be considered when enhancing implementation across different contexts.

Improving universal routine screening across CFIR domains of the implementation process requires attention to the relative importance that physicians place on screening features. Physicians emphasized test accuracy and efficiency—proximal factors central to the patient–clinician encounter—while placing less emphasis on more distal factors, such as operational tasks (screening location, type, and modality), which are typically managed by administrative support staff. Recognizing this distinction directs implementation efforts toward physician priorities on accuracy and time, while leveraging administrative staff to manage operational tasks.

To design an effective universal adolescent depression screening program, it is crucial to weigh preferences in the context of a joint decision-making framework involving patients, parents, and physicians.^70,71,72,73^ Future research should explore adolescent patient-centered or family-centered preferences^74,75^ and compare them with physician, administrative staff, or systems-centered preferences, while accounting for conjoint scale heterogeneity^76,77,78,79^ and mental care access considerations (eg, families with limited income) relevant to screening implementation.^80,81^ Family-centered conjoint studies^23,24^ have been successfully applied to improve pediatric primary care.^82,83^

### Limitations

This study has limitations. One study limitation is that our physician sample was not nationally representative. What physicians report they prefer and what they actually do in clinical practice may also not align. Survey responses may be subject to self-report bias or framing effects.^37^ While this study identified and valued the 5 key attributes that describe universal depression screening, other attributes beyond these may also influence physicians’ decision-making but were not assessed in this study. Our study focused on depression identification and diagnosis rather than treatment and management, but we recognize that enhancing care along the entire continuum of adolescent mental health delivery is crucial. We did not ask additional demographic questions or questions assessing attitudes or satisfaction about screening programs.

## Conclusions

In this survey study containing a DCE, top physician preferences were to avoid false negatives in depression screening, shorten the clinical appointment time, administer a 3-minute screener (PHQ-9), provide a private screening area, and use an electronic modality. Further research on family-centered preferences is needed to better understand priorities of adolescents and their families to improve access to and engagement with pediatric primary mental health care systems. Primary care physicians are strongly positioned to identify and treat adolescent depression early, and health systems and payers should consider physician preferences that could improve the implementation of national screening guidelines and strengthen the accuracy and efficiency of primary health systems for addressing pediatric mental health concerns to benefit children and families.

## Notes

Supplement 1. **eTable 1.** Sample Discrete Choice Question **eTable 2.** AAPOR Survey Reporting Checklist **eAppendix.** Evaluating Pediatrician Preferences on Implementing Universal Routine Screening of Adolescents for Major Depressive Disorder

Supplement 2.
Data Sharing Statement
