# Supplementary material for: Physician Preferences for Universal Routine Depression Screening for Adolescents in Primary Care
Source: JAMA Netw Open. 2025 Nov 25;8(11):e2545361. doi: 10.1001/jamanetworkopen.2025.45361 (PMC12648346; doi:10.1001/jamanetworkopen.2025.45361)
Supplement: Supplement 1. — eTable 1. Sample Discrete Choice Question eTable 2. AAPOR Survey Reporting Checklist eAppendix. Evaluating Pediatrician Preferences on Implementing Universal Routine Screening of Adolescents for Major Depressive Disorder [file jamanetwopen-e2545361-s001.pdf]

Supplemental Online Content

Doan TT, Wright DR, DeJonckheere M, Hutton DW, Ray KN, Prosser LA. Physician preferences for universal routine depression screening for adolescents in primary care. *JAMA Netw Open*. 2025;8(11):e2545361. doi:10.1001/jamanetworkopen.2025.45361

eTable 1. Sample Discrete Choice Question
eTable 2. AAPOR Survey Reporting Checklist
eAppendix. Evaluating Pediatrician Preferences on Implementing Universal Routine Screening of Adolescents for Major Depressive Disorder

This supplemental material has been provided by the authors to give readers additional information about their work.

© 2025 Doan TT et al. *JAMA Network Open*.

eTable 1. Sample Discrete Choice Question

**Attribute**	**Choice Profile A**	**Choice Profile B**	
Screening Modality	Paper	Paper	
Private Area to Complete Screener	No	No	
Screening Completion Time	3 minutes	10 minutes	
Missed Cases of Depression	10%	5%	
Allotted Clinician's Time	30 minutes	60 minutes	
If you had to choose one scenario, which would you prefer?	**Prefer A** ☐	**Prefer B** ☐	**Opt-out** ☐

© 2025 Doan TT et al. *JAMA Network Open*.

eTable 2. AAPOR Survey Reporting Checklist

**Checklist Item**	**Response**
1. Data Collection Strategy	The research team developed and validated a survey instrument containing a discrete choice experiment (DCE) in *Sawtooth Software*. This survey was administered online through a Qualtrics link with an embedded Sawtooth link (containing the DCE). Qualtrics LLC collected the survey data from a physician sample panel that they operate and then provided non-identified survey data responses to the research team.
2. Who Sponsored the Research and Who Conducted It	University of Michigan and University of Pittsburgh
3. Measurement Tools/Instruments	Quantitative survey instrument containing a discrete choice experiment (See Appendix Survey Instrument)
4. Population Under Study	Survey respondents were U.S. primary care physicians and were eligible to participate if they practiced in pediatrics, family medicine, or internal medicine and regularly saw adolescent patients aged 12-21 years as part of clinical practice.
5. Method Used to Generate and Recruit the Sample	Qualtrics LLC, a survey research firm, was contracted to administer the survey instrument to a national sample of primary care physicians, a respondent panel that is recruited and maintained by Qualtrics.<br><br>Using Qualtrics, survey data was collected efficiently and shared without identifiable information. Respondents were invited by email and underwent a validation process to verify themselves as medical professionals.<br><br>Consistent with AAPOR reporting guidelines, because the sample was recruited from an opt-in nonprobability panel, the participation rate cannot be reported because the sampling frame was unknown.
6. Method(s) and Mode(s) of Data Collection	Internet survey
7. Dates of Data Collection	Final survey instrument was administered to final sample from April to June 2024.
8. Sample Sizes	The final sample included 181 physician respondents who completed "high-quality" survey responses. An additional 117 responses were completed but determined to be of inadequate quality based on a priori rules (see Item #10 below) and, therefore, not included in data analysis.
9. How the Data Were Weighted.	No weighing.

© 2025 Doan TT et al. *JAMA Network Open*.

10. How the Data Were Processed and Procedures to Ensure Data Quality.	Qualtrics was paid $58 for each high-quality, completed physician survey response. Qualtrics excluded inadequate quality survey responses and replaced these respondents by inviting a new participant, and therefore, there were no missing responses. Responses were determined to be of inadequate quality if completed in under two minutes, a pattern was detected across questions, percentages of attribute importance were illogical, or the direction of attribute preferences was illogical.
11. A General Statement Acknowledging Limitations of the Design and Data Collection.	All research has limitations, and we acknowledge the unmeasured errors associated with all forms of survey research.

© 2025 Doan TT et al. *JAMA Network Open*.

Skip to:

eAppendix. Evaluating Pediatrician Preferences on Implementing Universal Routine Screening of Adolescents for Major Depressive Disorder

University of Pittsburgh

INFORMATION SHEET

You are invited to participate in a research study: "Evaluating pediatrician preferences on implementing universal routine screening of adolescents for major depressive disorder."

This project serves as part of original research performed by Dr. Tran Doan, PhD MPH, a General Academic Pediatrics Post-doctoral Fellow in the University of Pittsburgh's School of Medicine. This study has been approved by the University of Pittsburgh's Institutional Review Board for Human Subject Research (#23020197).

The following information will describe the steps involved in participating in the study, in order to help determine whether you are able to participate.

Please review the following information pertaining to this study:

1) The objective of this study is to understand what pediatricians think would best increase the universal depression screening rate in adolescents.
2) You will be asked to complete a survey, which is administered by Qualtrics. **The survey should take 10 minutes or less to complete.** Survey questions will include your preferences and experiences regarding universal adolescent depression screening in the clinical setting.
3) About 170 people will complete this study.
4) The survey will include some demographic questions about you and your practice. However, **you will not be identifiable as an individual,** and any potential risks to your privacy and/or confidentiality will be minimal.
5) This study does not directly benefit you. Information revealed from this survey could help pediatric clinicians integrate adolescent behavioral health care into their own practices.
6) This survey is completely voluntary. You can choose to stop taking the survey at any time.
7) Compensation will occur through Qualtrics, LLC (and not the study team). Participants will not receive compensation for incomplete responses.
8) If, at any time, you have questions about your rights as a research participant, or if you have questions, concerns, or other feedback about the survey, you may contact: doantt@upmc.edu.

By clicking, "Next Page" you certify that you have read and agree to the terms described above.

Back Next

0% 100%

© 2025 Doan TT et al. *JAMA Network Open*.

Skip to:

About 1 in 5 adolescents experience depression. The American Academy of Pediatrics recommends all teen patients should be screened annually for major depression.

I will describe 5 different features of a potential universal adolescent depression screening program:

- Screening modality
- Private area to complete screener
- Screening completion time
- Missed cases of depression
- Allotted clinician's time

Then, I will ask you to consider potential screening strategies with different combinations of these features.

Back Next

0% 100%

© 2025 Doan TT et al. *JAMA Network Open*.

Skip to:

This section describes 5 features of a universal adolescent depression screening program and the possible options for each feature. This is just for review.

Screening modality

Depression screener version can be administered using paper or electronic forms.

PAPER **ELECTONIC**

Private area to complete screener

Teen patients can be provided with a private area to complete the screener alone.

YES **NO**

Back Next

0% 100%

© 2025 Doan TT et al. *JAMA Network Open*.

Skip to:

This section describes 5 features of a universal adolescent depression screening program and the possible options for each feature. This is just for review.

Screening completion time

Depression screening tools vary in terms of how long they take to fill out.

**30 seconds**	**3 minutes**	**10 minutes**

Missed cases of depression

Screening tools vary in terms of how good they are at identifying accurate depression cases.

**25% of depressed teens remain undiagnosed.**	**10% of depressed teens remain undiagnosed.**	**5% of depressed teens remain undiagnosed.**

Back Next

0% 100%

© 2025 Doan TT et al. *JAMA Network Open*.

Skip to:

This section describes 5 features of a universal adolescent depression screening program and the possible options for each feature. This is just for review.

Allotted clinician time

This is the average length of time that a primary care clinician is allotted to spend on each adolescent patient, including scheduled appointment time and allotted documentation or administrative time.

**30 minutes**	**45 minutes**	**60 minutes**

Back Next

0% 100%

© 2025 Doan TT et al. *JAMA Network Open*.

Skip to:

Think about the AFOREMENTIONED features of a universal adolescent depression screening program.

Choose between two HYPOTHETICAL options.

• Imagine which of these options would best increase the adolescent depression screening rate.

• Imagine that you are making this decision for your own primary care practice.

• **Assume that your primary care practice has adequate resources for referral options and other mental specialty care.**

Back Next

0% 100%

© 2025 Doan TT et al. *JAMA Network Open*.

Skip to:

This is a PRACTICE question.

Imagine you are asked to make recommendations on how to best increase the adolescent depression screening rate.

Based on these features, **which option would you prefer for your practice?** You can also choose to opt out if you prefer neither. This is just for practice.

(1 of 1)

	Option 1	Option 2	
**Screening modality**	Paper	Paper	NONE: I wouldn't choose either.
**Private area to complete screener**	No	No	
**Screening completion time**	30 seconds	30 seconds	
**Missed cases of depression**	5% of depressed teens remain undiagnosed.	25% of depressed teens remain undiagnosed.	
**Allotted clinician time**	30 minutes	30 minutes	
	Select	Select	Select

Back Next

0% 100%

© 2025 Doan TT et al. *JAMA Network Open*.

Skip to:

Nice! Looks like you have the hang of this. Click Next to start the survey.

Screening modality	Paper	Paper
Private area to complete screener	No	No
Screening completion time	30 seconds	30 seconds
Missed cases of depression	5% of depressed teens remained undiagnosed.	25% of depressed teens remained undiagnosed.
Allotted clinician time	30 minutes	30 minutes
	Select	Select

NONE: I wouldn't choose either.

Select

Back Next

0% 100%

© 2025 Doan TT et al. *JAMA Network Open*.

Skip to:

The options on the left and right look similar, but the option on the right would lead to more missed depression cases. Review all of the attributes in each option as you go through the questions in the survey.

Screening modality	Paper	Paper
Private area to complete screener	No	No
Screening completion time	30 seconds	30 seconds
Missed cases of depression	5% of depressed teens remained undiagnosed.	25% of depressed teens remained undiagnosed.
Allotted clinician time	30 minutes	30 minutes
	Select	Select

NONE: I wouldn't choose either.
Select

Back Next

0% 100%

© 2025 Doan TT et al. *JAMA Network Open*.

Skip to:

The next questions will follow the same format.

• Read each of the options carefully.

• Consider all of the information given.

• There are a total of 13 questions.

• We understand that some of these questions may be difficult to answer. There are no right or wrong answers, we are just interested in hearing your opinions!

Back Next

0% 100%

© 2025 Doan TT et al. *JAMA Network Open*.

Skip to: [dropdown]

Imagine you are asked to make recommendations on how to best increase the adolescent depression screening rate. Based on these features, **which option do you prefer for your practice?** You can also choose NONE if you prefer neither.

(1 of 13)

	Option 1	Option 2	Option 3
**Screening modality**	Electronic	Paper	NONE: I wouldn't choose either.
**Private area to complete screener**	No	Yes	
**Screening completion time**	3 minutes	3 minutes	
**Missed cases of depression**	10% of depressed teens remain undiagnosed.	25% of depressed teens remain undiagnosed.	
**Allotted clinician time**	30 minutes	45 minutes	
	Select	Select	Select

Back Next

0% 100%

© 2025 Doan TT et al. *JAMA Network Open*.

Skip to:

Imagine you are asked to make recommendations on how to best increase the adolescent depression screening rate. Based on these features, **which option do you prefer for your practice?** You can also choose NONE if you prefer neither.

(2 of 13)

	Option 1	Option 2	Option 3
**Screening modality**	Electronic	Paper	NONE: I wouldn't choose either.
**Private area to complete screener**	Yes	Yes	
**Screening completion time**	30 seconds	10 minutes	
**Missed cases of depression**	25% of depressed teens remain undiagnosed.	10% of depressed teens remain undiagnosed.	
**Allotted clinician time**	30 minutes	45 minutes	
	Select	Select	Select

Back Next

0% 100%

© 2025 Doan TT et al. *JAMA Network Open*.

Skip to: [dropdown]

Imagine you are asked to make recommendations on how to best increase the adolescent depression screening rate. Based on these features, **which option do you prefer for your practice?** You can also choose NONE if you prefer neither.

(3 of 13)

	Option 1	Option 2	NONE
**Screening modality**	Electronic	Paper	NONE: I wouldn't choose either.
**Private area to complete screener**	No	No	
**Screening completion time**	3 minutes	30 seconds	
**Missed cases of depression**	5% of depressed teens remain undiagnosed.	25% of depressed teens remain undiagnosed.	
**Allotted clinician time**	60 minutes	45 minutes	
	Select	Select	Select

Back Next

0% 100%

© 2025 Doan TT et al. *JAMA Network Open*.

Skip to: [ ]

Imagine you are asked to make recommendations on how to best increase the adolescent depression screening rate. Based on these features, **which option do you prefer for your practice?** You can also choose NONE if you prefer neither.

(4 of 13)

	Option 1	Option 2	
**Screening modality**	Paper	Electronic	NONE: I wouldn't choose either.
**Private area to complete screener**	Yes	Yes	
**Screening completion time**	10 minutes	30 seconds	
**Missed cases of depression**	5% of depressed teens remain undiagnosed.	25% of depressed teens remain undiagnosed.	
**Allotted clinician time**	60 minutes	30 minutes	
	Select	Select	Select

Back Next

0% 100%

© 2025 Doan TT et al. *JAMA Network Open*.

Skip to:

Imagine you are asked to make recommendations on how to best increase the adolescent depression screening rate. Based on these features, **which option do you prefer for your practice?** You can also choose NONE if you prefer neither.

(5 of 13)

	Option 1	Option 2	NONE
**Screening modality**	Paper	Electronic	NONE: I wouldn't choose either.
**Private area to complete screener**	Yes	No	
**Screening completion time**	10 minutes	3 minutes	
**Missed cases of depression**	10% of depressed teens remain undiagnosed.	25% of depressed teens remain undiagnosed.	
**Allotted clinician time**	30 minutes	60 minutes	
	Select	Select	Select

Back Next

0% 100%

© 2025 Doan TT et al. *JAMA Network Open*.

Skip to:

Imagine you are asked to make recommendations on how to best increase the adolescent depression screening rate. Based on these features, **which option do you prefer for your practice?** You can also choose NONE if you prefer neither.

(6 of 13)

	Option 1	Option 2	
**Screening modality**	Electronic	Paper	NONE: I wouldn't choose either.
**Private area to complete screener**	No	No	
**Screening completion time**	30 seconds	3 minutes	
**Missed cases of depression**	10% of depressed teens remain undiagnosed.	5% of depressed teens remain undiagnosed.	
**Allotted clinician time**	60 minutes	45 minutes	
	Select	Select	Select

Back Next

0% 100%

© 2025 Doan TT et al. *JAMA Network Open*.

Skip to:

Imagine you are asked to make recommendations on how to best increase the adolescent depression screening rate. Based on these features, **which option do you prefer for your practice?** You can also choose NONE if you prefer neither.

(7 of 13)

	Option 1	Option 2	
**Screening modality**	Electronic	Paper	NONE: I wouldn't choose either.
**Private area to complete screener**	Yes	Yes	
**Screening completion time**	10 minutes	30 seconds	
**Missed cases of depression**	5% of depressed teens remain undiagnosed.	10% of depressed teens remain undiagnosed.	
**Allotted clinician time**	30 minutes	45 minutes	
	Select	Select	Select

Back Next

0% 100%

© 2025 Doan TT et al. *JAMA Network Open*.

Skip to:

Imagine you are asked to make recommendations on how to best increase the adolescent depression screening rate. Based on these features, **which option do you prefer for your practice?** You can also choose NONE if you prefer neither.

(8 of 13)

	Option 1	Option 2	Option 3
**Screening modality**	Paper	Electronic	NONE: I wouldn't choose either.
**Private area to complete screener**	No	Yes	
**Screening completion time**	30 seconds	3 minutes	
**Missed cases of depression**	25% of depressed teens remain undiagnosed.	5% of depressed teens remain undiagnosed.	
**Allotted clinician time**	60 minutes	45 minutes	
	Select	Select	Select

Back Next

0% 100%

© 2025 Doan TT et al. *JAMA Network Open*.

Skip to:

Imagine you are asked to make recommendations on how to best increase the adolescent depression screening rate. Based on these features, **which option do you prefer for your practice?** You can also choose NONE if you prefer neither.

(9 of 13)

	Option 1	Option 2	NONE
**Screening modality**	Electronic	Paper	NONE: I wouldn't choose either.
**Private area to complete screener**	No	No	
**Screening completion time**	30 seconds	10 minutes	
**Missed cases of depression**	25% of depressed teens remain undiagnosed.	10% of depressed teens remain undiagnosed.	
**Allotted clinician time**	45 minutes	30 minutes	
	Select	Select	Select

Back Next

0% 100%

© 2025 Doan TT et al. *JAMA Network Open*.

Skip to:

Imagine you are asked to make recommendations on how to best increase the adolescent depression screening rate. Based on these features, **which option do you prefer for your practice?** You can also choose NONE if you prefer neither.

(10 of 13)

	Option 1	Option 2	
**Screening modality**	Paper	Paper	NONE: I wouldn't choose either.
**Private area to complete screener**	Yes	Yes	
**Screening completion time**	3 minutes	3 minutes	
**Missed cases of depression**	5% of depressed teens remain undiagnosed.	25% of depressed teens remain undiagnosed.	
**Allotted clinician time**	30 minutes	60 minutes	
	Select	Select	Select

Back Next

0% 100%

© 2025 Doan TT et al. *JAMA Network Open*.

Skip to:

Imagine you are asked to make recommendations on how to best increase the adolescent depression screening rate. Based on these features, **which option do you prefer for your practice?** You can also choose NONE if you prefer neither.

(11 of 13)

	Option 1	Option 2	
**Screening modality**	Electronic	Electronic	NONE: I wouldn't choose either.
**Private area to complete screener**	No	Yes	
**Screening completion time**	10 minutes	30 seconds	
**Missed cases of depression**	5% of depressed teens remain undiagnosed.	25% of depressed teens remain undiagnosed.	
**Allotted clinician time**	60 minutes	45 minutes	
	Select	Select	Select

Back Next

0% 100%

© 2025 Doan TT et al. *JAMA Network Open*.

Skip to:

Imagine you are asked to make recommendations on how to best increase the adolescent depression screening rate. Based on these features, **which option do you prefer for your practice?** You can also choose NONE if you prefer neither.

(12 of 13)

	Option 1	Option 2	
**Screening modality**	Paper	Electronic	NONE: I wouldn't choose either.
**Private area to complete screener**	No	Yes	
**Screening completion time**	10 minutes	10 minutes	
**Missed cases of depression**	10% of depressed teens remain undiagnosed.	10% of depressed teens remain undiagnosed.	
**Allotted clinician time**	60 minutes	30 minutes	
	Select	Select	Select

Back Next

0% 100%

© 2025 Doan TT et al. *JAMA Network Open*.

Skip to:

Imagine you are asked to make recommendations on how to best increase the adolescent depression screening rate. Based on these features, **which option do you prefer for your practice?** You can also choose NONE if you prefer neither.

(13 of 13)

	Option 1	Option 2	NONE
**Screening modality**	Paper	Paper	NONE: I wouldn't choose either.
**Private area to complete screener**	Yes	No	
**Screening completion time**	10 minutes	30 seconds	
**Missed cases of depression**	5% of depressed teens remain undiagnosed.	10% of depressed teens remain undiagnosed.	
**Allotted clinician time**	45 minutes	30 minutes	
	Select	Select	Select

Back Next

0% 100%

© 2025 Doan TT et al. *JAMA Network Open*.

Skip to:

You finished all 13 choice questions! You're almost done. To wrap up the survey, please complete these demographic questions.

With which gender do you most identify?

- ◯ Male
- ◯ Female
- ◯ Gender-variant or gender-nonconforming
- ◯ Prefer not to disclose
- ◯ Prefer to self-disclose ______

What is your ethnicity?

- ◯ American Indian or Alaskan Native, not of HIspanic orgin
- ◯ Asian or Asian American, not of HIspanic orgin
- ◯ Black or African American, not of HIspanic orgin
- ◯ Hispanic, Latino, or of Spanish orgin
- ◯ Middle Eastern, not of HIspanic orgin
- ◯ Native Hawaiian or Other Pacific Islander, not of HIspanic orgin
- ◯ White or Caucasian, not of HIspanic orgin
- ◯ Mixed (please specify) ______
- ◯ Other (please specify) ______

What type of geographic setting do you practice in? (Check all that apply.)

- ☐ Urban
- ☐ Suburban
- ☐ Rural

Which region of the country do you primarily practice in?

- ◯ Northeast (Connecticut, Maine, Massachusetts, New Hampshire, New Jersey, New York, Pennsylvania, Rhode Island, and Vermont)
- ◯ Midwest (Illinois, Indiana, Iowa, Kansas, Michigan, Minnesota, Missouri, Nebraska, North Dakota, Ohio, South Dakota, and Wisconsin)
- ◯ South (Alabama, Arkansas, Delaware, District of Columbia, Florida, Georgia, Kentucky, Louisiana, Maryland, Mississippi, North Carolina, Oklahoma, South Carolina, Tennessee, Texas, Virginia, and West Virginia)
- ◯ West (Alaska, Arizona, California, Colorado, Hawaii, Idaho, Montana, Nevada, New Mexico, Oregon, Utah, Washington, and Wyoming)

In which primary care specialty area do you primarily practice?

- ◯ Pediatrics
- ◯ Family Medicine
- ◯ Internal Medicine
- ◯ Other (please specify) ______

What type of medical setting do you practice in? (Check all that apply.)

- ☐ Academic hospital
- ☐ Community health center
- ☐ Private practice

Back Next

0% 100%

© 2025 Doan TT et al. *JAMA Network Open*.

Skip to:

Thank you for taking this survey. You will now be redirected back to Qualtrics survey.

Note:

When respondents take the survey in regular mode this page will not be displayed. Respondents will be redirected to the url below:

https://pitt.co1.qualtrics.com/jfe/form/SV_8HOlwSR7TPBXByK?t=2&ID1= [Script] **&ID2=** [Script] **&ID3=** [Script] **&ID4=** [Script] **&ID5=** [Script] **&ID6=** [Script]

0% 100%

© 2025 Doan TT et al. *JAMA Network Open*.
