# Supplementary material for: Physician Preferences for Universal Routine Depression Screening for Adolescents in Primary Care
Source: JAMA Netw Open. 2025 Nov 25;8(11):e2545361. doi: 10.1001/jamanetworkopen.2025.45361 (PMC12648346; doi:10.1001/jamanetworkopen.2025.45361)
Supplement: Supplement 2. — Data Sharing Statement [file jamanetwopen-e2545361-s002.pdf]

Data Sharing Statement

Doan. Physician Preferences for Universal Routine Depression Screening for Adolescents in Primary Care. *JAMA Netw Open*. Published November 25, 2025.
doi:10.1001/jamanetworkopen.2025.45361

Data
Data available: Yes
Data types: Deidentified participant data
How to access data: Deidentified data is available upon request to the corresponding author with appropriate IRB and data use agreements in place.
When available: With publication

Supporting Documents
Document types: None

Additional Information
Who can access the data: Deidentified data is available upon request to the corresponding author with appropriate IRB and data use agreements in place.
Types of analyses: For research purposes.
Mechanisms of data availability: With investigator support after approval of a proposal and signed data access agreement
